# Targeting the GFI1/1B—CoREST Complex in Acute Myeloid Leukemia

**DOI:** 10.3389/fonc.2019.01027

**Published:** 2019-10-09

**Authors:** Maaike G. J. M. van Bergen, Bert A. van der Reijden

**Affiliations:** Laboratory of Hematology, Department of Laboratory Medicine, Radboud Institute for Molecular Life Sciences, Radboud University Medical Center, Nijmegen, Netherlands

**Keywords:** acute myeloid leukemia, histone modifications, KDM1A/LSD1 inhibitors, GFI1, GFI1B, CoREST, RCOR, HDAC1/2

## Abstract

One of the hallmarks of acute myeloid leukemia (AML) is a block in cellular differentiation. Recent studies have shown that small molecules targeting Lysine Specific Demethylase 1A (KDM1A) may force the malignant cells to terminally differentiate. KDM1A is a core component of the chromatin binding CoREST complex. Together with histone deacetylases CoREST regulates gene expression by histone 3 demethylation and deacetylation. The transcription factors GFI1 and GFI1B (for growth factor independence) are major interaction partners of KDM1A and recruit the CoREST complex to chromatin in myeloid cells. Recent studies show that the small molecules that target KDM1A disrupt the GFI1/1B–CoREST interaction and that this is key to inducing terminal differentiation of leukemia cells.

## Introduction

**Figure 1:**
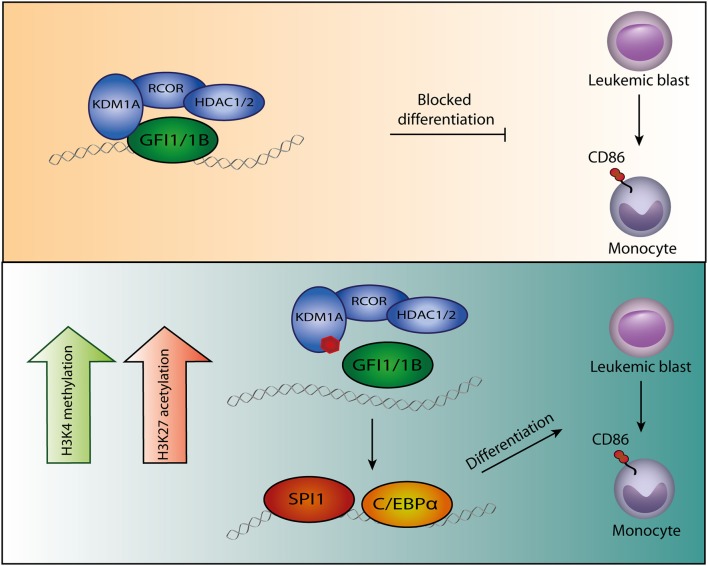
Model of GFI1/1B-KDM1A inhibition. By interacting with transcription factors GFI1/1B, the CoREST complex is recruited to DNA. **(Upper panel)** The CoREST complex catalyzes demethylation of histone marks H3K4me1/H3K4me2 and H3K9me1/H3K9me2, resulting in chromatin modifications and altered gene expression. The complex is stabilized by RCOR, which also facilitates HDAC1/2 binding. The HDACs contribute to gene repression by H3K9ac and H3K27ac deacetylation. In acute myeloid leukemia cells, GFI1/1B-CoREST contribute to a block in monocytic differentiation. **(Lower panel)** Small molecules (indicated by the red polygon) bind to FAD and inhibit the function of KDM1A as well as the interaction with transcription factors GFI1/1B. The release of GFI1-CoREST from chromatin allows binding of the myeloid transcription factors SPI1 and C/EBPα resulting in gene expression that forces the malignant cells to differentiate toward monocytes, exemplified by CD86 expression.

AML is a clonal heterogeneous disorder, characterized by a block in differentiation and increased proliferation of hematopoietic stem- and progenitor cells. Over the past years there have been many insights in the molecular pathogenesis of AML, that have been translated into new possibilities for targeted therapies (1). The biological processes underlying the growth advantage of leukemic stem cells, cell differentiation, and apoptosis are of major interest. Cellular changes are often associated with altered epigenetic modifications, that can be initiated by deregulated transcription factors (2). Epigenetic modifications are interesting targets, as they are often reversible. Currently, general therapies targeting the epigenome such as Azacytidine and Decitabine are being used widely for the treatment of myeloid malignancies (3). However, there is an urgent demand for more specific drugs targeting the biological processes underlying myeloid malignancies. The CoREST complex is an important epigenetic complex in hematopoietic development, of which its core components have been linked to malignant transformation (4–8). Core components of CoREST are KDM1A, histone deacetylases (HDACs) and REST corepressor family (RCOR) proteins (9–11). KDM1A binds histone 3 (H3) to demethylate di- and mono-methyl groups on lysine 4 and 9 (K4 and K9, respectively) while HDACs de-acetylate for instance H3K27 (12–15). In myeloid cells, the CoREST complex is recruited to chromatin via an interaction of KDM1A with the homologous transcription factors GFI1/1B (Figure 1). Small molecules inhibiting the function of KDM1A in myeloid malignancies are now emerging the field. In this review we will discuss the molecular function of the GFI1/GFI1B CoREST complex, and describe how targeting of this complex forces myeloblasts to differentiate at the molecular and cellular level and how this could improve treatment of leukemia.

## GFI1, GFI1B and the CoREST Complex in Myeloid Blood Cell Development

Transcription factors play a dominant role in controlling blood cell proliferation and differentiation. Two key transcription factors in these processes are GFI1 and GFI1B. Originally, GFI1 was identified in a screen for factors promoting interleukin-2 independent growth of a leukemia T-cell line, demonstrating that GFI1 plays an important role in cell proliferation (16). Later studies showed that GFI1 is important for lymphoid as well as myeloid differentiation whereas GFI1B is essential for the differentiation of megakaryocytes and erythrocytes (17–21). GFI1/1B are also crucial for the emergence of blood stem cells during murine embryogenesis and in adult mice they inhibit stem cell cycling (22–24). Inherited mutations in men have underscored the importance of GFI-proteins in blood cell development. Dominant-negative missense mutations in the DNA binding region of GFI1 result in an increase in monocyte numbers and block in granulocytic differentiation causing congenital neutropenia, characterized by recurrent infections (25, 26). Heterozygous, truncating germline mutations in the DNA binding domain of the parolog GFI1B cause autosomal dominant bleeding disorders. These are associated with macro-thrombocytopenia, hypogranular platelets, platelet CD34 expression, as well as increased proliferation of megakaryocytes in the bone marrow (27–31). Although direct targets in the disease pathogenesis of mutant GFI1B remain unknown, it was shown that genes implicated in blood coagulation were not properly induced (32). Thus, GFI1/1B control the growth and differentiation of different blood cell lineages.

GFI proteins have distinct functions in blood cell development, yet have a highly similar structure. Both transcription factors contain six C-terminal zinc fingers and a conserved N-terminal Snail/GFI1 (SNAG) domain (33–37). The intermediary domain between the SNAG domain and zinc fingers is different and the function is largely unknown. GFI1/1B bind DNA through zinc fingers 3-5 (35). Through their SNAG domain, the proteins bind the CoREST complex via an interaction with one of its core components KDM1A (Figure 1). Since its discovery, many CoREST associated proteins have been identified but the exact role of these components in myeloid biology remains largely unknown (38).

In line with the KDM1A-GFI1/1B interaction, KDM1A by itself plays an important role in blood cell development as well as hematopoietic stem cell (HSC) self-renewal capacity and the emergence of hematopoietic stem cells (39, 40). KDM1A contains a catalytic amine oxidase (AO) domain, which upon association with its co-factor flavine-adenine dinucleotide (FAD) demethylates H3K4 and H3K9. KDM1A knockout (KO) or knockdown (KD) mice show a severe terminal maturation defect in red blood cell and granulocyte development and platelet generation, but not in monocyte maturation. In addition, an increase in myeloid progenitor cells and mature HSC was observed (39, 41). In line with its demethylase activity, deletion of KDM1A resulted in increased H3K4me1 and H3K4me2 levels, which should normally be demethylated on hematopoietic stem and progenitor genes during differentiation.

KDM1A is composed of three main structures, a SWIRM domain (Swi3p, Rsc8p, and Moira), a Tower domain, and the above mentioned catalytic amine AO domain. The SWIRM domain associates with the AO domain to form a hydrophobic groove that allows KDM1A to bind H3 tails. The Tower domain provides a binding site on KDM1A for RCORs (42, 43). Major functions of the RCORs are preventing KDM1A from proteasomal degradation, stabilization of the complex and recruitment of the HDACs (44). There are three REST corepressor family members (RCOR1, RCOR2, and RCOR3) and they all interact with KDM1A. They have a common ELM2 (egl-27 and MTA homology2) domain, and two SANT (Swi3, Ada2, N-CoR, and TFIIIB) domains (45). The C-terminal SANT domain interacts with KDM1A to form a tight protein complex (44, 46). The ELM2 domain interacts with the N-terminal SANT domain to establish binding of HDAC1 and 2 (9, 47, 48). Depending on cell context, RCOR1 and RCOR2 enhance the demethylase function of KDM1A, while RCOR3 may counteract its activity, especially during erythrocyte and megakaryocyte differentiation (49, 50). HDAC1 and HDAC2 are highly similar proteins which bind the CoREST complex via an interaction with the RCORs. Besides being part of CoREST, they are also present in many other protein complexes. HDACs repress gene expression by removing active histone acetyl marks, such as H3K27ac. Deletion of *Hdac1* and *Hdac2* in mice resulted in cell cycle arrest, as well as apoptosis of megakaryocytes accompanied by thrombocytopenia, similar to *Gfi1b* deletions (24, 51). Although HDACs bind the CoREST complex via an interaction with the RCORs, it has been suggested that they directly interact with GFI1/1B as well (35).

GFI1/1B repress gene expression through CoREST recruitment to DNA. The SNAG domain and the interaction with KDM1A are crucial for GFI1/1B biology. A single point mutation at position two (P2A) in this domain that abrogates KDM1A binding renders wild type GFI1/1B completely inactive in neutrophil differentiation and inhibition of megakaryoblast growth, respectively (32, 52). Moreover, the same point mutation renders a dominant-negative bleeding disorder GFI1B mutation completely inactive (32). In addition to the proline at position two, a lysine at position eight within the SNAG domain needs to be dimethylated for efficient KDM1A binding (53). Similar to the P2A mutation, a K8A point mutation rendered GFI1B completely inactive in erythrocyte differentiation and loss of the interaction between KDM1A and GFI1B was observed. Interactions between GFI1/1B-CoREST have been confirmed by co-immunoprecipitation and chromatin immunoprecipitation experiments showing major overlap in GFI1/1B, KDM1A and RCOR1 binding (32, 35, 54–58). Thus, the CoREST interaction through KDM1A is crucial for GFI1/1B to control blood cell specification.

Finally, KDM1A has been shown to demethylate non-histone proteins such as TP53 which is mediated through GFI1 by TP53 binding (59). Dimethylation of K372 of TP53 activates TP53, whereas its monomethylation represses TP53 (60, 61). GFI1 KO mice have increased levels of K372 methylated TP53, resulting in TP53 activation and accelerated myeloid cell death (62, 63). Taken together, GFI1/1B interplay with KDM1A and the CoREST complex to regulate gene expression and to inhibit TP53 activity.

## GFI1, GFI1B, and the CoREST Complex in Myeloid Malignancies

GFI1/1B control the growth and differentiation of myeloid cells, and disruption of this function may contribute to the development and maintenance of AML cell expansion (35, 63–66). Remarkably, both low and high GFI1/1B expression have been implicated in malignant myeloid cell development. For instance, significantly increased *GFI1* expression was observed in cells derived from AML patients carrying the oncofusion RUNX1-RUNX1T1 (67). Ablation of GFI1 expression in RUNX1-RUNX1T1 mice models delayed AML initiation and progression, defining GFI1 as an oncogene in this AML subtype. However, KD, but also forced expression of GFI1/1B in human leukemia cell lines inhibited their growth and induced apoptosis (32, 68). In a megakaryoblast leukemia cell line, it was shown that GFI1B induced growth inhibition strictly depended on the KDM1A interacting P2 and K8 in GFI1B's SNAG domain (32). In mice, both *Gfi1* KD and KO resulted in increased immature myeloid cell numbers which transformed to a myeloproliferative disease in *Gfi1* KD but not KO mice (63). In this study, it was shown that complete *Gfi1* absence resulted in impaired KDM1A-mediated TP53 demethylation resulting in TP53 activation and cellular apoptosis. In contrast, low GFI1 levels were sufficient to maintain KDM1A-mediated TP53 demethylation to inhibit TP53 function and apoptosis. In combination with inhibition of apoptosis, the increased production of reactive oxygen species (ROS) observed in GFI1 KD mice may contribute to the onset of myeloproliferative disorders that can progress into myeloid leukemia. The myeloid transformation could be rescued by restoring GFI1 expression, confirming that low GFI1 expression is oncogenic (63). Besides aberrant GFI1 expression, a polymorphism in the intermediary domain in *GFI1* (rs34631763, changing a serine at position 36 to an asparagine, S36N) predisposes to human AML development (69, 70). This may in part be caused by the inability of GFI1^36N^ to regulate *HOXA9* gene expression (71). Regarding GFI1B, a rare somatic missense variant in the DNA binding domain (D262N) was found in a patient upon transformation of myelodysplastic syndrome (MDS) to AML (72). The mutant functioned in a dominant-negative manner to inhibit erythroid- and stimulate myeloid cell survival, thereby potentially contributing to the observed transformation. This indicates that GFI1B functions as tumor suppressor. Finally, wild type GFI1B is highly expressed in cells from patients with chronic myeloid leukemia (CML) (73). CML is caused by the mutated, constitutively active tyrosine kinase BCR-ABL1. Treatment with BCR-ABL1 tyrosine kinase inhibitors results in specific CML cell death. In part, this is caused by a strong GFI1B induction that in turn represses anti-apoptotic *BCL-xL* expression (74). Thus, in this setting GFI1B functions also as a tumor suppressor. Together, the studies mentioned above show that depending on cellular context, expression levels and the presence of variants, GFI1/1B may function as tumor suppressor or as oncogene, either by directly altering gene expression or by inhibiting TP53 activity. It will be interesting to determine in what conditions (high vs. low expression) pharmacological GFI1/1B inhibition is an effective anti-cancer strategy.

When investigated, the biological role of GFI1/1B did depend on an intact SNAG domain strongly suggesting that the interaction with KDM1A is key to malignant transformation. In line with these findings clear roles for the CoREST components KDM1A and HDACs have been identified in myeloid malignancies (4, 75, 76). In an AML mouse model positive for the MLL-AF9 oncofusion protein, KDM1A contributed to the inhibition of cell differentiation. KDM1A bound at similar regions as MLL-AF9 to maintain expression of an oncogenic gene program (5). Furthermore, aberrant recruitment of HDACs in leukemia by oncogenic fusion proteins such as RUNX1-RUNX1T1 and PML-RAR contributes to disease pathogenesis (6). Whether this strictly relies on the CoREST complex or other complexes in which HDAC1 and 2 are core complexes remains to be seen. Targeting the CoREST complex remains of major interest in the development of AML therapeutics. Inhibitors targeting KDM1A have been developed in recent years, and several clinical trials have started to test small molecules inhibiting KDM1A.

## KDM1A Inhibitors Induce Forced Monocytic Differentiation of Malignant Myeloblasts

High KDM1A expression has been implicated in cancer development and cancer progression (5, 8, 39, 41, 75, 76). Based on these findings, KDM1A has been defined as therapeutic target. An important class of KDM1A inhibitors are represented by tranylcypromine (TCP) derivatives (77). TCP was originally designed as an antidepressant targeting flavin dependent monoamine oxidases A and B (MAO-A, MAO-B). It binds irreversibly to FAD, that upon binding inhibits the AO domain (78). Based on sequence homology of the AO domain between MAO-A/B and KDM1 proteins, inhibition of the latter by TCP was demonstrated (79). Through this inhibition, KDM1A can no longer remove methyl groups from K4 and K9 of histone 3 (Figure 1). Initially, a KDM1A inhibitor was successfully combined with all-trans retinoic acid (ATRA) to induce differentiation of AML cells that are insensitive to ATRA alone (80). Exposure of 165 cancer cell lines to another inhibitor showed a strong effect on AML and small cell lung cancer (SCLC) cell lines, but hardly any effect on other cell types (81). This has been followed by the development and validation of numerous TCP-derivatives (Table 1) (77). Treatment of leukemia cell lines with KDM1A inhibitors promoted monocytic differentiation (exemplified by CD86 expression) and subsequent cell death at the nanomolar range (89). Terminal differentiation was observed of cell lines representing myeloblasts, megakaryoblasts and erythroblasts (Table 1). Especially, the latter two exhibited transdifferentiation toward monocytic-like cells upon KDM1A inhibition (86, 88) (Table 1). Primary human blasts also differentiated upon KDM1A inhibitor exposure and clear responses were observed in AML mice models. Based on these results, several KDM1A inhibitors have been or are being tested in clinical trials (Table 1).

Table 1Overview of CoREST inhibitors tested *in vitro* or *in vivo*.

**Table:** 

**Inhibitor**	**GSK2879552**	**GSK-LSD1**	**IMG-7289**	**NCD38**	**OG86**	**ORY-1001**	**T-3775440**	**TCP**
Clinical trial	NCT02177812, NCT02929498	n.a.	NCT02842827	n.a.	n.a.	EudraCT 2013-002447-29	n.a.	NCT02273102 NCT02717884 NCT02261779
Study model	165 cancer cell lines tested, 20/29 AML cell lines were sensitive^a^, various primary AML samples	Various AML cell lines^a^, MEG-01, SET2, MV4;11, primary MLL-AF9 xenografts, various primary AML samples	SET2 cells, Mice with JAK2 V617F positive MPN^b^	Primary MDS and AML samples, MV4;11, KPLRY, MDS-L, SKM-1, HL60, HEL and CMK cells	Various AML cell lines, e.g., THP1, MV4;11, HL60, K562, NB4^c^, murine MLL-AF9 AML cells, primary human MLL translocated leukemia samples	24 AML cell lines^d^, MV4;11 xenograft mice and two AML patients	AEL and AMKL xenograft mice models, TF-1a, HEL92.1.7, CMK11-5, M07e	Various AML cell lines (HL-60, TEX, U937, KG1a)^c^, primary AML samples
Increased CD86 expression	Yes	Yes	n.a.	n.a.	Yes	Yes	Yes	Yes
Histone modifications	n.a.	Global gain chromatin accessible sites (ATAC-seq) Increased H3K27ac and H3K4me2	Global increase in H3K4me3 and H3K9me2	Gain in H3K4me3 and H3K27ac but not in H3K4me2 at the GFI1 promoter.	No significant increase in H3K4me1 and H3K4me2, significant increase in H3K4me3, H3K9ac, and H3K27ac	Increased H3K4me2 at selected target genes	Increased H3K4me2 at KDM1A target PI16	Global increase H3K4me2
Affecting GFI1/1B KDM1A interaction?	n.a.	Yes	n.a.	Yes	Yes	Yes^e^	Yes	n.a.

**Table:** 

**Inhibitor**	**Major findings**	**References**
GSK2879552	*In vitro:* In total 20/29 AML cell lines were sensitive to the treatment. Reduced colony growth of AML cells and differentiation of cells. Combined treatment with ATRA^f^ increased growth reduction in AML cell line, associated with enrichment of differentiation associated gene signatures in AML cells. Increased caspase mediated cell death when the drugs were combined, as well as impaired colony formation.	(81–83)
GSK-LSD1	*In vitro*: Differentiation of AML cells toward the myeloid lineage, associated with increase in granule formation, nuclear condensation and maturation of the cells. Accumulation of cells in G0-G1 phase, suggesting cell cycle arrest of AML cells. Decreased differentiation toward megakaryocytes. Combination treatment with azacytidine was highly sensitive in TET2 mutated AML.	(32, 54, 58, 81–83)
	*In vivo:* In mice a decrease of MLL-AF9 positive cells was observed, associated with decline in platelet count.	
IMG-7289	*In vitro:* Decreased expression of BCL-xL (anti-apoptosis), cell cycle arrest, decreased proliferation, decreased colony formation which was stronger when combined with JAK-inhibitor Ruxolitinib. *In vivo:* Improved blood cell counts, reduced spleen volumes, restored splenic architecture, reduced bone marrow fibrosis. Inhibited proliferation of the cells, induced apoptosis of JAK2 V617F cells. Increased expression and methylation of TP53 and PUMA, decreased expression of BCL-xL.	(84)
NCD38	*In vitro:* Myeloid differentiation and block in cell growth in HEL, CMK and MDS-L cells. Derepression of super-enhancers from hematopoietic regulators [such as GFI1 and ETS-related gene (ERG)], inducing a myeloid development program.	(85, 86)
OG86	*In vitro:* Loss of clonogenic potential in cell lines and primary human MLL-AF9 AML cells. Increase in differentiation of the cells, associated with increased expression of myloid genes (SPI1, C/EBPα). Increase in CFU-M potential but less erythroid colonies. *In vivo:* In mice xenografts less circulating MLL-translocated AML cells were observed, which was accompanied with anemia and thrombocytopenia.	(5, 55)
ORY-1001	*In vitro:* Induction of differentiation markers in THP1 cells associated with macrophage/monocytic differentiation. Reduced colony forming potential was observed. Cell lines carrying the MLL-AF9 fusion were most sensitive to ORY-1001 treatment, whereas BCR-ABL1, PML-RARA, RUNX1-RUNX1T1 positive cell lines were less sensitive to the treatment, although still significantly affected. *In vivo:* AML xenograft mice models showed a reduction in percentage of leukemic blasts after treatment, as well as increased survival.	(87)
T-3775440	*In vitro:* Transdifferentiation of megakaryocyte and erythrocyte leukemic cell lines toward myeloid lineage. Transcriptional derepression of GFI1B target genes. *In vivo:* In AEL^g^/AMKL^h^ xenograft mice a rapid tumor decrease as well as platelet number reduction was observed.	(88)
TCP	*In vitro:* TCP KDM1A inhibition led to ATRA driven responses in non-APL AML. Increased expression of myeloid differentiation associated genes. *In vivo*: Diminished engraftment of primary human AML cells (FAB M1^i^) in NOD-SCID^j^ mice in combined treatment with ATRA.	(5, 80)

*n.a,not available*.

a For more information see Smitheman et al. (82).

b MPN: myeloproliferative neoplasms.

c For more information see Harris et al. (5).

d For more information see Maes et al. (87).

e GFI1/1B KDM1A interaction was diminished but not absent.

f ATRA; all -trans retinoic acid.

g Acute erythroblast leukemia.

h Acute megakaryoblast leukemia.

i FAB M1: French-American-British classification Acute myeloblastic leukemia without maturation.

j NOD-SCID mice: Nonobese diabetic severe combined immunodeficient mice.

Chromatin immunoprecipitation data in myeloblasts showed a major overlap between GFI1/1B, KDM1A and RCOR1 binding at enhancers of key myeloid differentiation genes (54–56, 58). Inhibition of KDM1A in myeloblasts resulted in increased expression of these genes (83) (Table 1). These changes were accompanied by a gain in chromatin accessible sites, generally associated with increased H3K4 methylation (in line with KDM1A inhibition), but also an increase in H3K27ac levels was observed. A motif analysis of emerging chromatin accessible regions showed enrichment for SPI1, C/EBPα and RUNX2 binding sites (54, 56, 58). SPI1 showed increased binding to these regions following KDM1A inhibition (54). KD experiments targeting SPI1 or deleting C/EBPα showed that KDM1A inhibition was no longer effective in inducing myeloid differentiation (54). This suggests that upon KDM1A inhibition, SPI1 and C/EBPα take over to initiate a myeloid differentiation program (Figure 1).

## Small Molecule Disruption of the KDM1A-GFI1/1B Interaction Is Key to Myeloid Cell Differentiation

The SNAG domain shows remarkable similarity to the N-terminal region of H3. SNAG domain peptides bind the KDM1A catalytic AO region in a very similar fashion as those representing the tail of histone 3 (90). In fact, SNAG-peptide KDM1A interactions are stronger than those of H3K4me. Binding of TCP-FAD adducts to KDM1A inhibit the GFI1/1B interaction (Table 1, Figure 1) (32, 55, 58, 86, 88). As CoREST requires an interaction with transcription factors to be recruited to chromatin, disruption of this interaction could be important for terminal differentiation. Indeed, GFI1 KD in a myeloblast cell line had a similar effect on gene expression as KDM1A inhibition by inducing a myeloid differentiation program (55, 85). Moreover, forced expression of a GFI1-KDM1A fusion protein blocked terminal differentiation upon inhibitor exposure, indicating that the separation between these proteins is biologically relevant (55). In the myeloblast cell line studied, the catalytic activity of KDM1A appeared non relevant for the maintenance of its clonogenic potential. This conclusion was based on the fact that impaired growth following KDM1A KD was corrected by re-introducing wild type but also catalytically inactive KDM1A. In an independent study, similar results were observed in the context of an erythroblast cell line (58). Separation of GFI1B and KDM1A appeared essential for drug induced differentiation and KDM1A function in malignant transformation did not depend on its catalytic activity. Together, these results might suggest that the catalytic activity of KDM1A in the CoREST-GFI1/1B complex is less important than its scaffold function in leukemogenesis. Also because KDM1A inhibition did not result in significant increased H3K4me (in contrast to several other studies, Table 1) but rather increased H3K27ac (55). Based on these findings it was concluded that increased H3 acetylation, rather than increased H3K4 methylation is important for differentiation. In contrast, in another recent study it was shown that complete loss of KDM1A and expression of a catalytic inactive mutant of KDM1A resulted in differentiation of murine myeloid leukemia cells. Thus, the block in differentiation mediated by KDM1A did not solely depend on its scaffold function toward GFI1 (56). *In vivo* KO of KDM1A in murine leukemia cells resulted in prolonged survival, while expression of catalytic inactive KDM1A did not prolong survival. Based on these findings it was concluded that both the scaffold function and the catalytic activity of KDM1A need to be inhibited for effective leukemia treatment. The observed differences in H3K4-methylation following KDM1A inhibition in published studies may be caused by differences in treatment duration and timepoints of measurements. Furthermore, differences in the used model systems (human vs. murine models, cell lines vs. transduced cells) and the use of different inhibitors may play a role in this as well. Additional studies in which normal KDM1A is replaced by catalytically inactive KDM1A, followed by use of the inhibitors may give a more clear result as to whether enzymatic activity vs. separation of GFI1/1B from KDM1A are both therapeutically relevant.

## Targeting the KDM1-RCOR Interaction

Besides FAD targeting KDM1A inhibitors, a reversible inhibitor that inhibits the binding of KDM1A to RCOR has been designed (SP2509, Table 1). Treatment of myeloblasts with the inhibitor showed increased H3K4 and H3K9 methylation (91). The compound inhibited growth of leukemia cell lines and primary blasts and induced differentiation. A combination treatment with the HDAC inhibitor Panobinostat caused synergistic effects in cell killing compared to either drug alone. Although these results are encouraging, it remains to be seen whether they can be attributed to KDM1A inhibition alone as the drug exhibited identical cytotoxic activities in normal and KDM1A null HAP1/BCR-ABL1+ cells (92).

## Future Perspectives

The inhibition of KDM1A has emerged as a potential treatment for AML as this forces malignant cells to terminally differentiate. KDM1A is a lysine specific demethylase that targets mono- and dimethylated histone 3 as well as other cellular proteins. H3K27 deacetylation and H3K4 demethylation both contribute to a repressive chromatin state leading to inhibition of gene expression. The best studied KDM1A inhibitors are the antidepressant TCP and derivatives thereof. By binding the catalytic active amine oxidase pocket, these drugs inhibit the demethylase activity of KDM1A toward histone 3. In myeloid cells, KDM1A is one of the core components of the CoREST complex. This complex is recruited to chromatin through the transcription factors GFI1/1B. Binding of aforementioned inhibitors to KDM1A block the interaction between KDM1A and GFI1/GFI1B. This triggers the dissociation of KDM1A and GFI1 from chromatin and subsequently decreases repressive marks and increases activating marks. The induction of a myeloid gene expression program that follows, triggers the differentiation of AML cells irrespective of their nature; both megakaryoblasts, erythroblasts and myeloblasts are forced to differentiate upon inhibition of GFI1/GFI1B. This is somewhat counterintuitive, as GFI-proteins and KDM1A normally stimulate myeloid, erythroid and megakaryocytic differentiation and this depends on the SNAG domain that interacts with KDM1A. Apparently, GFI1/1B-CoREST adopt other functions in malignant transformation that can be counteracted by their inhibition. Alternatively, GFI1/1B may exclusively block monocytic differentiation which can be overcome by KDM1A inhibition.

In two independent studies, it was shown that in THP1, SET2, and MV4;11 leukemia cells the separation of GFI1/1B from KDM1A rather than inhibiting its enzymatic activity played a crucial growth inhibitory role (55, 58). However, KDM1A is part of several other chromatin complexes besides CoREST that do not contain GFI1/1B (77). For instance, in breast cancer cells KDM1A associates with the zinc finger protein ZNF516, which together regulate the expression of the epidermal growth factor receptor (EGFR). Dysregulation of this receptor has been implicated in malignant transformation (93). Thus, it remains to be seen whether KDM1A inhibition affects other processes than CoREST associated functions that may contribute to cell differentiation. Apart from the TCP-like inhibitors other types of KDM1A inhibitors have been developed [for an extensive review see (77)]. Recently, a dual inhibitor that simultaneously inhibits KDM1A and class I HDACs was developed (94). This inhibitor targets CoREST more efficiently and induced cell death more effective than single HDAC inhibition in melanoma. It will be interesting to determine whether the dual inhibitor exhibits enhanced activity in AML.

KDM1A also demethylates non-histone proteins such as TP53. GFI1 facilitates TP53 demethylation resulting in its inhibition. Although most studied myeloid leukemia cell lines have inactive TP53, it is conceivable that KDM1A inhibition contributes to TP53 activation and apoptosis in primary AML. This is relevant because in the majority of AML cases, TP53 is not mutated and it can be re-activated (95). KDM1A also binds as pseudosubstrate to the tumor suppressor and ubiquitin ligase FBXW7 (96). This results in FBXW7 auto-ubiquitination and subsequent proteasomal and lysosomal degradation. Because this function of KDM1A is independent of its catalytic activity it will be important to determine whether inhibitor induced release from chromatin will allow KDM1A to diminish FBXW7 levels and whether this counteracts cell death.

GFI1/1B are methylated in their SNAG domain on K8 and this methylation is important for efficient KDM1A recruitment and function (53, 97). Whether KDM1A/CoREST can remove this methyl mark followed by dissociation from GFI1/1B, and subsequent demethylation of nearby histone 3, remains an open question. Yet, an increase in H3 methylation and acetylation following KDM1A inhibition at regions bound by GFI1/1B have been observed, although these findings were not seen in all studies. This inconsistency is likely due to different AML models, inhibitors and duration of the treatment used. Besides MLL-AF9 positive leukemia, studies targeting different AML subtypes are pointing toward a broader purpose for KDM1A inhibition in leukemia development. Recent insights have shown that different AML subtypes are sensitive to treatment with a KDM1A inhibitor (83, 98). The RUNX1-RUNX1T1 translocation was among the most sensitive subtypes, which might be explained by the essential role of GFI1 in the maintenance of the leukemic cell growth (67). Interestingly, a recent study showed that the combination of Azacytidine with KDM1A inhibition has an enhanced effect on targeting leukemic stem cells compared to either treatment individually (83). Particularly primary TET2 mutated AML cells were sensitive to this combination, suggesting novel approaches to sensitize less-responsive AML subtypes.

Finally, we and others showed that many proteins associate with CoREST in the context of GFI1/1B (32, 35, 57). Although the effect on CoREST function has been studied for some of these factors, the role of others like ZNF217, and PHF21A is still unclear. Taken together, further (clinical) studies are required to answer these outstanding questions, and determine the most efficient approach to induce cellular differentiation of the different leukemia subtypes.

## Author Contributions

MB performed the literature search and wrote the manuscript together with BR.

### Conflict of Interest

The authors declare that the research was conducted in the absence of any commercial or financial relationships that could be construed as a potential conflict of interest.
